# Global, regional, and national burden of gastroesophageal reflux disease (1990–2021): age-period-cohort analysis and Bayesian projections

**DOI:** 10.3389/fpubh.2025.1576527

**Published:** 2025-07-09

**Authors:** Liumei Mo, Zhenhao Liu, Wenjing Cao, Hanxian Gong, Jincheng Wu, Mingzhuo Lin, Wei Pan

**Affiliations:** ^1^Department of Geriatrics, The Affiliated Foshan Women and Children Hospital, Guangdong Medical University, Foshan, China; ^2^Department of Cardiovascular Medicine, Pingxiang People's Hospital, Pingxiang, China; ^3^Ganzhou Traditional Chinese Medicine Hospital, Ganzhou, China; ^4^The Eighth Affiliated Hospital of Southern Medical University (The First People’s Hospital of Shunde), Foshan, China

**Keywords:** gastroesophageal reflux disease, GBD, age-standardized rate, age-period-cohort, Bayesian projections

## Abstract

**Background:**

Gastroesophageal reflux disease (GERD) is a common chronic upper gastrointestinal disorder that causes discomfort and increases the risk of esophageal adenocarcinoma. The global burden of GERD has steadily increased, establishing it as a critical public health issue. This study systematically evaluated the global, regional, and national burden of GERD from 1990 to 2021, revealing epidemiological trends and regional disparities to inform targeted intervention strategies.

**Methods:**

We utilized data from the 2021 Global Burden of Disease Study (GBD) to assess the prevalence, incidence, and years lived with disability (YLDs) of GERD. Key indicators included age-standardized prevalence rate (ASPR), age-standardized incidence rate (ASIR), age-standardized YLDs rate (ASYR), and estimated annual percentage change (EAPC). Analyses were stratified by age, gender, and socio-demographic index (SDI). Age-period-cohort (APC) models were applied to examine trends in the 15–49 age group, and Bayesian APC models were used to project future trends.

**Results:**

In 2021, global GERD prevalence reached 825.6 million (95% uncertainty interval: 732.99–925.56 million). From 1990 to 2021, incident cases, prevalent cases, and YLDs rose by 83.16, 80.06, and 82.46%, respectively. The ASIR, ASPR, and ASYR all showed upward trends, with EAPCs of 0.097, 0.076, and 0.083. The highest burden of ASIR, ASPR, and ASYR was observed in Tropical Latin America in 2021, while the lowest was in East Asia. Regions with lower SDI had higher ASIR, ASPR, and ASYR. In the 15–49 age group, the GERD burden increased with age across all SDI regions, with unfavorable period effects observed in high and high-middle SDI regions, as well as adverse cohort effects in high-middle and middle SDI regions. Projections indicate that by 2035, global GERD cases in this age group will reach 527.2 million (ASPR: 12,082.06/100,000), with 214.6 million incident cases (ASIR: 4,916.68/100,000) and 4.1 million YLDs (ASYR: 94.47/100,000).

**Conclusion:**

GERD poses a growing global health challenge. Insights from these observed epidemiological patterns can assist policymakers in developing targeted measures to reduce its impact, particularly in high-risk regions and younger demographics.

## Introduction

Gastroesophageal reflux disease (GERD) is a prevalent condition of the upper gastrointestinal tract, characterized by various symptoms and potential complications due to the backward flow of stomach contents into the esophagus, pharynx (throat), or lungs. Common clinical manifestations include acid regurgitation and heartburn. In advanced cases, it may cause serious complications, including ulcers, esophagitis, strictures, and a heightened risk of developing esophageal adenocarcinoma (1). The chronic and recurring nature of GERD greatly diminishes the patient’s quality of life and presents significant economic challenges for individuals, families, healthcare systems, and society. The disease often requires prolonged medical care, contributing to the extensive utilization of healthcare and social resources (2, 3). Recently, the global incidence and prevalence of GERD have steadily increased, underscoring its significance as a growing public health issue (4, 5).

Recent systematic reviews have analyzed the trends in the incidence and prevalence of GERD over the past few decades (6, 7). The Global Burden of Disease (GBD) 2019 study also provided a comprehensive evaluation of GERD’s global burden (5, 8). Despite these efforts, GERD’s burden has demonstrated temporal evolution, with significant disparities across and within regions and nations. Recently, factors, including improved living standards, shifts in lifestyle and dietary patterns, rising rates of overweight and obesity, and an aging population, have driven the increasing incidence and prevalence of GERD (9, 10). These dynamic changes underscore the necessity of a systematic, consistent, and comparative analysis of GERD trends at regional and national levels. This approach is crucial for guiding global intervention strategies.

The GBD study, one of the largest epidemiological projects, provides comprehensive data on the incidence, prevalence, and disability rates of 371 diseases across various demographics (11). Utilizing data from the 2021 GBD study, this research systematically evaluated GERD’s burden globally and regionally from 1990 to 2021, with a particular focus on the effects of age, period, and cohort on disease trends in individuals aged 15–49, and forecasted the future burden for this group (12). This study aimed to provide insights for global healthcare policy and resource allocation, addressing the growing GERD burden and promoting public health interventions.

## Methods

### Data sources

This research leveraged data from the 2021 GBD study, which provided comprehensive estimates of health loss from 371 diseases, injuries, and conditions, and 88 risk factors across 204 countries and 811 subnational regions between 1990 and 2021 (11). The 2021 GBD study aggregated epidemiological data from diverse sources, including national health surveys, hospital records, mortality databases, and peer-reviewed literature. These datasets were processed using advanced, standardized methods to generate reliable and comparable estimates of health metrics, facilitating robust analyses across global, regional, and national levels.

GERD-related data were extracted using the Global Health Data Exchange query tool^1^. GERD diagnoses were categorized based on the International Classification of Diseases, 10th Revision, using codes R12 11, K21–K21.9, and K22.7–K22.719 (11). The extracted variables included prevalent cases, years lived with disability (YLDs), incident cases, and their respective age-standardized rates across global, regional, and national levels. Each metric was accompanied by 95% uncertainty intervals (UIs), which were calculated based on the 25th and 975th ordered values of the posterior distribution from 1,000 draws (11).

Data were stratified by age (5–9 years, in 5-year intervals up to 95 + years), sex, calendar year (1990–2021), and geographic location (regions and countries). Globally, the data encompassed 21 GBD regions, and 204 countries and territories were grouped into quintiles according to socio-demographic index (SDI) (11). SDI, a composite measure of socio-economic development, combines indicators, including fertility rates, educational attainment, and income per capita, with scores ranging from 0 to 1. Lower SDI scores indicate lower levels of socio-economic advancement.

### Temporal trends and SDI in GERD burden

The estimation techniques employed by GBD 2021 have been described in previous studies. To estimate GERD burden metrics, including prevalence, YLDs and incidence, DisMod-MR 2.1, a Bayesian meta-regression framework, was utilized (11, 13).

The annual age-standardized prevalence rate (ASPR), age-standardized incidence rate (ASIR), and age-standardized YLDs rate (ASYR) for GERD were calculated globally, regionally, and nationally from 1990 to 2021. Temporal trends were analyzed using the estimated annual percentage change (EAPC), derived from a log-linear regression model where the natural logarithm of the age-standardized rate was regressed against time (year). As a commonly used measure for assessing changes in disease burden, EAPC values and their 95% confidence intervals (CIs) indicate trends: positive values suggest an upward trend, whereas negative values reflect a decline. Pearson correlation analysis was employed to examine the association between GERD burden metrics (ASIR, ASPR, and ASYR) and SDI, evaluating the influence of socio-economic factors on GERD. A positive correlation indicated that higher SDI scores were associated with a greater GERD burden, while a negative correlation suggested the opposite.

### APC model analysis

To analyze the dynamic changes in GERD burden by age, period, and birth cohort, we applied the APC model (14, 15). The APC model is a widely used tool in epidemiology that breaks down temporal trends into three main effects: age, period, and cohort. By disentangling these factors, the APC model provides a clearer understanding of how age, time period, and birth cohort independently influence disease burden.

In our study, we used the APC model to examine GERD trends, focusing on key factors like incidence, prevalence, and YLDs, particularly within the 15–49 age group. This demographic was selected because of growing apprehension about GERD among younger populations. The model helps identify variations in GERD burden across different age groups, sexes, and birth cohorts, offering valuable insights for public health planning. Furthermore, the model also examines the role of socio-economic factors, such as the SDI, in influencing GERD burden across age, birth cohorts and time periods. Overall, the APC model enhances our understanding of GERD, especially among younger people, and provides valuable insights into gaps in prevention, management, and treatment.

In this analysis, we used 5-year age groups aligned with 5-year periods. Data from the 2021 GBD database was utilized, including GERD prevalence, incidence, YLDs, and population data for individuals aged 15–49 from 1992 to 2021. The age groups were categorized as: 15–19, 20–24, 25–29, 30–34, 35–39, 40–44, and 45–49 years. The study periods were divided into six five-year intervals: 1992–1996, 1997–2001, 2002–2006, 2007–2011, 2012–2016, and 2017–2021. Additionally, the analysis included 12 overlapping 10-year birth cohorts. Although the overall analysis covers 1990 to 2021, APC modeling was conducted from 1992 onward to align with 5-year period groupings and ensure model consistency. The APC model estimated overall temporal trends and trends in prevalence, incidence, and YLDs for each age group. The overall trend was represented by net drift (annual percentage change), while the localized trend was represented by local drift, which describes the annual change rate within each age group. The significance of these trends was assessed using the Wald χ^2^ test.

In the APC model, the age effect is represented by age-specific rates, while period and cohort effects are quantified through relative risks. The relative risks were calculated by comparing each period or cohort’s age-specific rates to a selected reference group. The choice of the reference group did not affect the overall interpretation of the results, as it merely served as a baseline for comparisons.

### Prediction

To project the disease burden for the 15–49 age group from 2022 to 2035, we applied the BAPC model, implemented using the Integrated Nested Laplace Approximations (version 24.6.27) and BAPC (version 0.0.36) packages in R software (15, 16). This approach enabled us to forecast future disease burdens and provide evidence-based insights to policymakers on the projected GERD burden. These projections should be interpreted in light of the underlying assumptions of the Bayesian model, particularly in regions with limited historical data.

### Statistics

ASPR, ASIR, and ASYR were reported as the estimated rates per 100,000 population, along with their 95% UIs. All statistical analyses and graphical representations were conducted using R software (version 4.4.1). A two-tailed *p* < 0.05 was considered statistically significant. All analyses conducted as part of the GBD 2021 study followed the Guidelines for Accurate and Transparent Health Estimate Reporting.

### Ethics

The institutional review board exempted this study because it utilized publicly accessible data that did not include confidential or personally identifiable patient information.

## Results

### Trends in global and SDI-specific GERD burden

**Figure 1:**
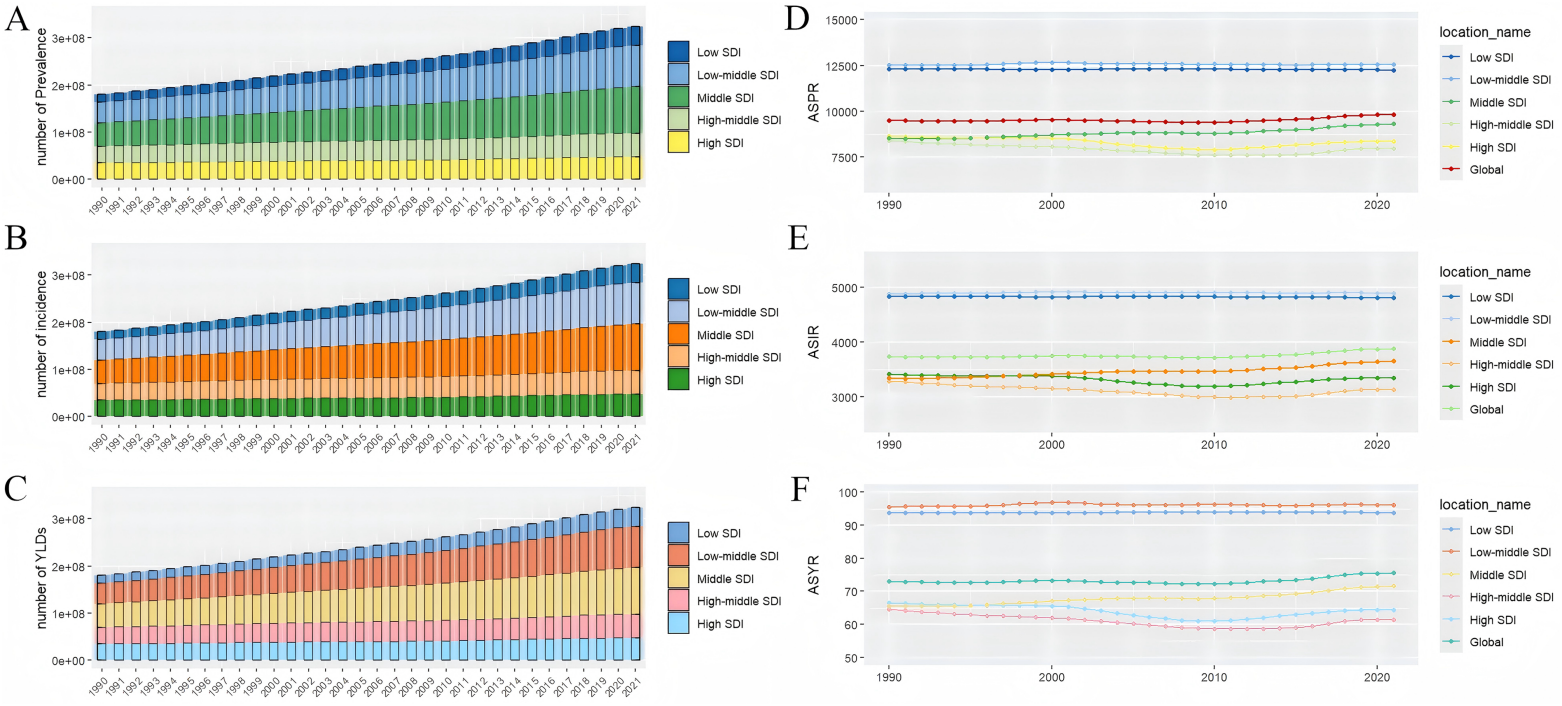
The prevalence numbers **(A)**, incidence numbers **(B)**, YLD numbers **(C)**, ASPR **(D)**, ASIR **(E)**, and ASYR **(F)** of GERD in global and the five SDI regions from 1990 to 2021. SDI, sociodemographic Index, ASPR; ASIR, age-standardized incidence rate; ASYR, age-standardized years lived with disability; GERD, gastroesophageal reflux disease. ASPR, age-standardized prevalence rate; ASIR, age-standardized incidence rate; ASYR, age-standardized years lived with disability; EAPC, estimated annual percentage change; GERD, gastroesophageal reflux disease.

From 1990 to 2021, the global burden of GERD continued to increase (Figures 1A–C). The number of GERD cases globally increased from approximately 450.77 million in 1990 (95% UI: 397.48 million–511.64 million) to about 826.6 million cases in 2021 (95% UI: 732.99 million–925.56 million), reflecting a growth of 83.16%. Furthermore, the ASPR of GERD rose from 9,516.49/100,000 population in 1990 (95% UI: 8,427.33–10,664.72) to 9,838.60/100,000 in 2021 (95% UI: 8,732.46–11,056.05), with an EAPC of 0.076 (95% CI: 0.035–0.117; Table 1; Supplementary Table 1). Besides, the number of incident GERD cases globally in 2021 was approximately 324.14 million (95% UI: 287.69 million–358.91 million), an increase of 80.06% from 180.02 million cases in 1990 (95% UI: 158.66 million–199.95 million). The ASIR increased from 3,739.86/100,000 in 1990 (3,314.20–4,142.33) to 3,881.86/100,000 in 2021 (3,445.56–4,299.95), with an EAPC of 0.097 (95% CI: 0.061–0.132; Table 1; Supplementary Table 2). In 2021, the global YLDs due to GERD reached approximately 6.34 million (95% CI: 1.12 million–3.19 million), representing an increase of 82.46% from 3.47 million in 1990 (95% CI: 1.75 million–6.13 million). The ASYR rose from 73.01/100,000 in 1990 (36.75–129.66) to 75.56/100,000 in 2021 (95% CI: 38.05–133.87), with an EAPC of 0.083 (95% CI: 0.043–0.123; Table 1; Supplementary Table 3).

**Table 1:** Prevalence, incidence, and YLDs for GERD in 1990 and 2021 across global, five SDI regions, and 21 GBD regions, with the EAPC from 1990 to 2021.

Location	Prevalence					Incidence					YLDs				
	All-age cases (95% UI) 1990	All-age cases (95% UI) 2021	ASRs per 100 000 population (95% UI) 1990	ASRs per 100 000 population (95% UI) 2021	EAPC in ASRs (95% CI) 1990–2021	All-age cases (95% UI) 1990	All-age cases (95% UI) 2021	ASRs per 100 000 population (95% UI) 1990	ASRs per 100 000 population (95% UI) 2021	EAPC in ASRs (95% CI) 1990–2021	All-age cases (95% UI) 1990	All-age cases (95% UI) 2021	ASRs per 100 000 population (95% UI) 1990	ASRs per 100 000 population (95% UI) 2021	EAPC in ASRs (95% CI) 1990–2021
Global	450765454.69 (397478515.38 to 511638409.85)	825603654.11 (732989499.88 to 925555127.88)	9516.49 (8427.33 to 10664.72)	9838.60 (8732.46 to 11056.05)	0.076 (0.035 to 0.117)	180018233.37 (158660995.15 to 199950073.37)	324139599.3 (287693228.7 to 358,912,516)	3739.86 (3314.20 to 4142.33)	3881.86 (3445.56 to 4299.95)	0.097 (0.061 to 0.132)	3472702.58 (1752692.84 to 6128627.19)	6336162.70 (11241352.50 to 3189793.94)	73.01 (36.75 to 129.66)	75.56 (38.05 to 133.87)	0.083 (0.043 to 0.123)
SDI quintile															
Low SDI	40756198.71 (35858366.27 to 46662089.50)	96970987.20 (85122154.68 to 111319921.83)	12301.93 (10923.72 to 13752.96)	12261.34 (10889.35 to 13711.04)	−0.008 (−0.010 to −0.005)	16607901.75 (14551684.7 to 18469681.04)	39536248.57 (34516600.96 to 44014348.38)	4829.002513 (4287.062419 to 5342.744925)	4809.962512 (4268.663825 to 5318.3446)	−0.010 (−0.012 to −0.007)	313170.23 (158640.01 to 552442.67)	748244.48 (378515.25 to 1323334.99)	93.72 (47.37 to 166.76)	93.73 (47.36 to 166.86)	0.009 (0.006 to 0.013)
Low-middle SDI	107823563.56 (95386761.99 to 122669881.87)	223640427.34 (198450231.05 to 253280672.97)	12526.50 (11151.33 to 14013.04)	12563.56 (11184.77 to 14064.75)	−0.002 (−0.015 to 0.010)	43396197.56 (38366263.24 to 48127012.67)	43396197.56 (38366263.24 to 48127012.67)	4882.264138 (4352.479012 to 5408.162815)	4894.018722 (4362.317361 to 5416.808182)	0.004 (−0.004 to 0.012)	829855.41 (419541.26 to 1471666.22)	1718835.43 (864434.02 to 3039007.72)	95.63 (48.12 to 169.56)	96.06 (48.29 to 169.97)	0.007 (−0.006 to 0.019)
Middle SDI	124042191.97 (108873698.26 to 141290274.85)	252457133.76 (223782410.73 to 282932217.10)	8528.06 (7566.43 to 9556.85)	9314.67 (8280.55 to 10467.81)	0.272 (0.244 to 0.300)	50019121.85 (43995351.08 to 55575248.45)	98388205.58 (87127604.58 to 108930034.4)	3345.955228 (2974.027994 to 3704.326979)	3660.464816 (3252.073951 to 4047.064356)	0.281 (0.256 to 0.307)	960429.52 (486151.54 to 1705950.47)	1940951.33 (974677.90 to 3452926.61)	65.54 (32.98 to 116.41)	71.61 (36.01 to 126.92)	0.275 (0.248 to 0.303)
High-middle SDI	90575890.68 (79428771.23 to 102076033.12)	131003975.07 (115415492.44 to 146131971.90)	8413.25 (7392.13 to 9436.87)	7968.92 (7053.35 to 8914.06)	−0.245 (−0.327 to −0.163)	35652655.2 (31443953.74 to 39686082.38)	50357993.28 (44527625.01 to 55976704.96)	3293.700182 (2900.964086 to 3661.820797)	3130.059174 (2771.29221 to 3484.45477)	−0.226 (−0.301 to −0.151)	697106.38 (352828.17 to 1237087.21)	1003165.10 (503937.25 to 1782283.89)	64.60 (32.61 to 114.71)	61.33 (31.01 to 108.81)	−0.233 (−0.315 to −0.152)
High SDI	87040987.10 (76439202.58 to 97176617.53)	120765088.77 (106344887.85 to 134868183.27)	8639.91 (7584.02 to 9703.44)	8379.95 (7363.75 to 9424.80)	−0.186 (−0.275 to −0.098)	34136778.55 (29922480.92 to 38124823.62)	47166281.07 (41524019.23 to 52774700.94)	3418.567016 (2986.044525 to 3815.08339)	3353.312762 (2926.719193 to 3742.912588)	−0.132 (−0.205 to −0.059)	668088.06 (336434.66 to 1193738.35)	919090.78 (462214.16 to 1632390.94)	66.45 (33.49 to 119.01)	64.36 (32.49 to 114.95)	−0.188 (−0.276 to −0.101)
Regions															
Andean Latin America	4806203.70 (4241223.51 to 5407619.34)	10891746.50 (9601460.37 to 12223517.60)	16409.79 (14499.06 to 18314.03)	16405.98 (14495.47 to 18306.62)	−0.001 (−0.001 to −0.001)	1856762.99 (1650176.58 to 2070989.0)	4072657.76 (3613213.53 to 4506116.09)	6101.73 (5438.31 to 6737.72)	6099.59 (5437.15 to 6734.2)	−0.001 (−0.001 to −0.001)	37339.36 (18866.02 to 66400.60)	84213.21 (42562.03 to 149397.34)	35.35 (17.72 to 62.91)	126.58 (64.01 to 223.80)	0.000 (−0.002 to 0.002)
Australasia	1960613.43 (1717239.27 to 2200757.82)	3398336.11 (2997307.44 to 3825173.57)	8779.29 (7656.61 to 9846.20)	8777.72 (7654.99 to 9845.29)	0.025 (−0.115 to 0.165)	787266.71 (687934.36 to 883727.64)	1346855.74 (1182680.16 to 1504797.01)	3542.28 (3088.2 to 3979.31)	3542.46 (3088.47 to 3978.8)	0.019 (−0.079 to 0.117)	15019.22 (7508.73 to 26634.16)	25916.06 (12978.00 to 45738.42)	42.32 (21.28 to 76.15)	67.44 (33.81 to 119.43)	0.026 (−0.115 to 0.168)
Caribbean	5131526.18 (4538133.50 to 5746829.60)	8405596.17 (7431550.85 to 9365567.27)	16412.31 (14502.11 to 18315.67)	16408.86 (14498.52 to 18308.49)	−0.001 (−0.001 to −0.001)	1953701.51 (1738949.35 to 2170054.33)	3106454.76 (2776439.49 to 3417854.66)	6102.11 (5438.55 to 6738.49)	6100.63 (5437.95 to 6736.16)	−0.001 (−0.001 to −0.001)	39715.78 (20191.57 to 70317.33)	64630.36 (32801.12 to 114011.36)	67.32 (33.70 to 119.49)	126.30 (63.98 to 223.15)	−0.005 (−0.006 to −0.003)
Central Asia	6226354.97 (5414310.73 to 7077815.96)	10393680.55 (9031140.30 to 11782472.78)	10880.72 (9507.41 to 12199.25)	10865.69 (9494.59 to 12187.79)	−0.004 (−0.004 to −0.004)	2523218.65 (2213209.3 to 2814204.36)	4133538.7 (3613385.38 to 4611899.13)	4321.79 (3813.97 to 4806.07)	4317.35 (3809.52 to 4802.35)	−0.003 (−0.003 to −0.002)	48180.51 (24506.35 to 84987.14)	80296.33 (40643.51 to 142347.72)	83.82 (42.39 to 148.06)	83.68 (42.19 to 147.61)	−0.000 (−0.002 to 0.001)
Central Europe	15476639.90 (13627518.06 to 17338174.36)	17487422.12 (15431232.85 to 19362916.57)	11102.53 (9781.19 to 12450.17)	11189.98 (9850.75 to 12545.83)	0.029 (0.028 to 0.031)	6033314.27 (5312670.75 to 6702640.36)	6664588.56 (5925379.49 to 7384056.31)	4363.7 (3842.38 to 4832.61)	4392.32 (3870.96 to 4864.31)	0.024 (0.023 to 0.025)	118476.39 (59909.37 to 210193.63)	133153.32 (66619.30 to 235606.57)	48.04 (23.99 to 86.70)	86.05 (43.35 to 152.64)	0.042 (0.040 to 0.045)
Central Latin America	20572735.00 (18206803.55 to 23126087.61)	43556882.36 (38559082.78 to 48562213.75)	16420.77 (14563.53 to 18283.32)	16429.42 (14570.23 to 18291.39)	0.002 (0.001 to 0.002)	8047123.2 (7150120.91 to 8954727.72)	16350512.69 (14578247.13 to 17895434.39)	6161.54 (5503.87 to 6741.05)	6162.98 (5505.46 to 6740.47)	0.000 (−0.000 to 0.001)	159476.41 (80804.43 to 282895.63)	335462.01 (169810.07 to 591600.70)	105.22 (53.30 to 185.75)	126.40 (64.00 to 222.56)	0.002 (−0.000 to 0.004)
Central Sub-Saharan Africa	4040853.06 (3501790.67 to 4645781.02)	10684957.29 (9257616.31 to 12353076.90)	11406.01 (10040.17 to 12841.34)	11403.42 (10039.70 to 12828.08)	−0.001 (−0.002 to −0.001)	1665153.02 (1454925.74 to 1868796.15)	4396283.33 (3812333.81 to 4954038.85)	4509.51 (3952.88 to 5031.98)	4508.19 (3950.9 to 5033.91)	−0.001 (−0.002 to −0.001)	30956.67 (15875.10 to 54937.97)	82407.80 (42004.45 to 146606.31)	85.14 (42.99 to 150.92)	87.09 (44.24 to 156.90)	0.023 (0.020 to 0.026)
East Asia	52564012.55 (45736394.44 to 60315334.53)	84447601.29 (73524441.41 to 95427845.72)	4572.35 (3982.87 to 5188.54)	4554.18 (3961.91 to 5170.75)	−0.082 (−0.216 to 0.053)	21657883.9 (18719586.44 to 24587864.72)	33637020.03 (28943253.6 to 38112786.07)	1856.21 (1621.35 to 2093.92)	1849.87 (1609.66 to 2090.47)	−0.076 (−0.199 to 0.046)	408903.68 (207380.23 to 721067.69)	650318.92 (323834.58 to 1159390.19)	88.93 (44.84 to 157.05)	35.22 (17.68 to 62.55)	−0.080 (−0.212 to 0.053)
Eastern Europe	30045170.44 (26367037.45 to 33738818.98)	31647395.88 (27779943.40 to 35404695.89)	11623.88 (10182.52 to 13060.68)	11593.98 (10161.10 to 13024.27)	−0.188 (−0.301 to −0.074)	11747714.1 (10345127.65 to 13074504.5)	12175725.11 (10796381.67 to 13579954.01)	4590.71 (4043.55 to 5114.67)	4580.24 (4035.48 to 5100.44)	−0.154 (−0.249 to −0.060)	229392.22 (116294.49 to 404122.78)	240365.17 (121615.44 to 426742.09)	128.31 (65.28 to 226.86)	88.73 (44.67 to 156.85)	−0.176 (−0.288 to −0.064)
Eastern Sub-Saharan Africa	13648432.59 (11904901.16 to 15652630.20)	33681826.59 (29396705.35 to 38843844.78)	11584.80 (10222.78 to 13043.05)	11595.51 (10229.70 to 13050.55)	0.002 (0.001 to 0.002)	5643894.08 (4932893.23 to 6306155.03)	13906524.56 (12066920.9 to 15614112.53)	4580.06 (4025.35 to 5095.97)	4583.75 (4027.78 to 5100.72)	0.001 (0.001 to 0.002)	105181.65 (53709.03 to 187068.13)	260625.45 (132632.91 to 465543.27)	126.32 (64.09 to 222.42)	88.84 (44.90 to 159.47)	0.022 (0.019 to 0.025)
High-income Asia Pacific	12320093.30 (10788472.55 to 13945332.66)	17064185.29 (14999947.39 to 19371777.29)	6215.47 (5439.05 to 7044.02)	6295.18 (5512.72 to 7133.68)	0.192 (0.085 to 0.300)	5029062.17 (4403132.72 to 5630395.24)	6777190.14 (5987675.54 to 7610830.24)	2550.17 (2231.67 to 2860.11)	2580.33 (2258.22 to 2887.89)	0.159 (0.073 to 0.245)	95194.03 (47498.16 to 171609.90)	130351.54 (64866.93 to 234919.42)	126.60 (64.09 to 223.80)	48.72 (24.42 to 88.16)	0.199 (0.091 to 0.307)
High-income North America	34065272.42 (29673615.51 to 38152668.12)	45181084.85 (39753843.46 to 50717140.77)	10691.09 (9279.78 to 12055.62)	9595.09 (8402.41 to 10842.16)	−0.627 (−0.832 to −0.420)	12982927.83 (11377720.82 to 14443654.61)	17494199.05 (15416218.08 to 19443438.21)	4111.1 (3588.35 to 4566.76)	3794.49 (3313.53 to 4242.52)	−0.483 (−0.653 to −0.313)	261040.75 (131505.59 to 464737.92)	341552.31 (174664.28 to 610200.75)	40.85 (20.78 to 73.58)	73.21 (37.26 to 131.48)	−0.644 (−0.847 to −0.440)
North Africa and Middle East	30763375.15 (26969036.11 to 35118971.17)	74657640.12 (66067413.58 to 84430574.62)	12322.04 (10870.21 to 13854.19)	12411.31 (11066.28 to 13825.27)	0.031 (0.010 to 0.052)	12433731.15 (10918819.08 to 13870452.16)	29265665.36 (25678681.28 to 32620732.47)	4819.92 (4253.79 to 5357.61)	4836.65 (4293.67 to 5351.47)	0.011 (−0.003 to 0.025)	238165.78 (119570.20 to 420995.21)	575185.22 (290196.61 to 1016801.27)	63.34 (31.88 to 112.98)	95.11 (47.96 to 167.82)	0.024 (0.004 to 0.045)
Oceania	254399.40 (220830.06 to 291921.77)	606910.75 (525939.56 to 695653.55)	5324.19 (4657.24 to 6015.72)	5325.80 (4658.08 to 6017.80)	−0.000 (−0.001 to 0.000)	106819.1 (92868.34 to 121220.99)	251635.57 (218734.17 to 285023.71)	2166.66 (1896.97 to 2442.25)	2167.33 (1897.19 to 2443.04)	−0.000 (−0.000 to 0.000)	1972.04 (1001.92 to 3494.31)	4700.72 (2394.27 to 8347.16)	86.60 (44.25 to 155.41)	40.87 (20.80 to 73.02)	0.003 (0.002 to 0.005)
South Asia	112724579.34 (99435800.11 to 128351644.39)	244459048.03 (216995403.16 to 276666339.48)	13662.32 (12177.79 to 15324.36)	13661.56 (12175.34 to 15313.74)	−0.010 (−0.021 to 0.001)	45304672.74 (39908976.11 to 50385397.14)	96465350.47 (84966662.55 to 107155382.93)	5323.55 (4733.69 to 5897.15)	5324.47 (4735.23 to 5897.01)	−0.003 (−0.010 to 0.004)	866274.87 (437196.18 to 1535860.54)	1875347.47 (946467.34 to 3330656.46)	94.69 (47.54 to 167.51)	104.28 (52.71 to 185.20)	0.001 (−0.011 to 0.012)
Southeast Asia	20320413.58 (17692749.52 to 23297618.83)	40428938.80 (35035890.29 to 45983577.44)	5495.07 (4810.98 to 6211.31)	5493.35 (4809.00 to 6212.23)	−0.002 (−0.002 to −0.002)	8438772.09 (7357716.71 to 9560854.17)	16344888.87 (14269226.13 to 18517056.73)	2223.19 (1956.69 to 2512.38)	2222.39 (1955.85 to 2511.31)	−0.002 (−0.003 to −0.002)	157800.10 (79765.03 to 280834.24)	312709.54 (156956.57 to 567268.69)	82.08 (41.32 to 146.78)	42.37 (21.29 to 76.29)	0.007 (0.006 to 0.009)
Southern Latin America	6517108.91 (5706866.90 to 7306985.37)	10564558.50 (9283510.54 to 11772718.64)	13658.09 (11963.22 to 15269.66)	13654.53 (11959.83 to 15264.82)	−0.140 (−0.192 to −0.088)	2392029.97 (2121244.0 to 2651093.87)	3816331.84 (3395847.19 to 4238885.12)	4986.72 (4419.15 to 5529.91)	4985.6 (4418.34 to 5528.66)	−0.080 (−0.110 to −0.050)	50244.81 (25491.07 to 88674.75)	81068.49 (41091.79 to 143141.64)	126.73 (64.01 to 224.19)	105.04 (53.17 to 186.43)	−0.141 (−0.193 to −0.088)
Southern Sub-Saharan Africa	4532148.33 (3978928.74 to 5151366.71)	8859981.62 (7748482.11 to 10077770.44)	11792.39 (10434.48 to 13212.95)	11804.55 (10447.92 to 13226.90)	0.003 (0.001 to 0.005)	1854646.15 (1624068.29 to 2067573.02)	3541989.8 (3089189.06 to 3975059.93)	4660.05 (4105.04 to 5187.63)	4663.54 (4108.83 to 5192.32)	0.002 (0.001 to 0.004)	34942.14 (17773.14 to 62044.04)	67673.73 (34309.45 to 121330.47)	88.48 (44.78 to 159.00)	89.67 (45.30 to 160.39)	−0.019 (−0.021 to −0.017)
Tropical Latin America	21565372.45 (19158540.23 to 24324205.78)	42699176.91 (37847552.71 to 47252480.46)	16769.60 (15002.03 to 18631.95)	16681.34 (14832.41 to 18433.23)	−0.093 (−0.122 to −0.064)	8310404.18 (7382342.68 to 9188869.79)	15834153.18 (14081470.84 to 17386189.99)	6270.12 (5623.81 to 6884.86)	6248.12 (5567.61 to 6874.8)	−0.048 (−0.062 to −0.035)	166145.82 (85285.20 to 295862.42)	326624.96 (165370.23 to 577471.95)	104.05 (52.57 to 184.62)	127.65 (64.69 to 225.33)	−0.088 (−0.115 to −0.061)
Western Europe	38418286.60 (33905722.01 to 42601095.67)	48522835.84 (42849078.65 to 54435997.43)	8233.13 (7253.55 to 9245.33)	8222.18 (7208.31 to 9249.61)	0.011 (0.002 to 0.020)	15173305.39 (13359262.53 to 16904747.62)	18917642.47 (16679273.72 to 21114806.35)	3297.99 (2874.87 to 3680.35)	3296.77 (2878.87 to 3692.37)	0.014 (0.004 to 0.024)	294227.10 (147919.11 to 519155.64)	369683.33 (185203.70 to 653462.38)	88.83 (44.87 to 159.65)	63.30 (31.76 to 112.67)	0.015 (0.005 to 0.024)
Western Sub-Saharan Africa	14811863.39 (12971587.25 to 16947241.12)	37963848.54 (33123174.88 to 43714343.95)	11622.96 (10248.90 to 13061.09)	11640.17 (10258.20 to 13083.01)	0.005 (0.005 to 0.006)	6075830.17 (5311324.2 to 6771689.58)	15640391.34 (13609464.02 to 17514451.47)	4595.96 (4043.06 to 5115.15)	4601.02 (4045.23 to 5122.3)	0.004 (0.003 to 0.004)	114053.25 (58087.02 to 203061.00)	293875.76 (149414.37 to 524437.57)	90.26 (45.53 to 161.60)	89.30 (45.08 to 160.52)	0.023 (0.021 to 0.026)

GBD, Global Burden of Disease; EAPC, estimated annual percentage change; CI, confidence interval; SDI, socio-demographic index; UI, uncertainty interval; ASR, age-standardized rate; YLDs, years of life lived with disability; GERD, gastroesophageal reflux disease.

From 1990 to 2021, the numbers of GERD prevalence, incidence, and YLDs continued to rise across all five SDI regions. In 2021, higher SDI levels were linked to lower GERD ASIR, ASPR, and ASYR. The values for the middle, high-middle, high, and SDI quintiles were below the global average, whereas those for the low-middle and low SDI quintiles were higher than the global rate (Table 1; Figures 1D–F). In 2021, the high-middle SDI quintile reported the lowest ASPR, ASIR, and ASYR. The ASPR exhibited a decreasing trend in the low, low-middle, high-middle, and high SDI quintiles, whereas it increased in the middle SDI quintile (Table 1; Figure 1D; Supplementary Table 1). ASIR declined in the low, high-middle, and high SDI quintiles from 1990 to 2021, although it rose in the low-middle and middle SDI quintiles (Table 1; Figure 1E; Supplementary Table 2). Although ASYR declined in the high-middle and high SDI quintiles, it increased in the middle, low, and low-middle SDI quintiles (Table 1; Figure 1F; Supplementary Table 3).

### GERD burden across GBD regions

**Figure 2:**
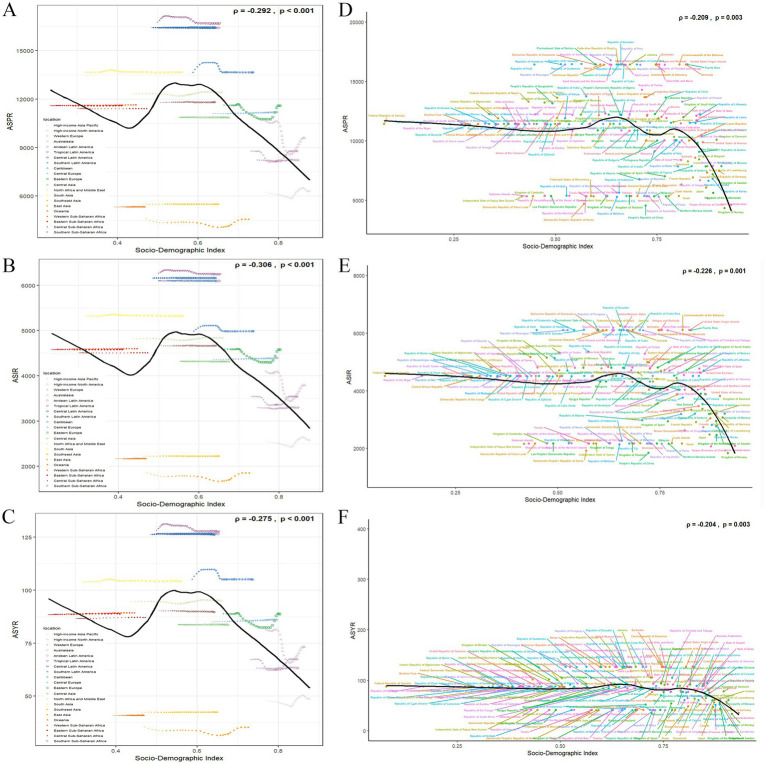
The ASPR **(A)**, ASIR **(B)**, and ASYR **(C)** due to GERD for 21 GBD regions by SDI from 1990 to 2021. The black line represents expected rates in 2021 based solely on SDI. Points from left to right show estimates from 1990 to 2021. The ASPR **(D)**, ASIR **(E)**, and ASYR **(F)** due to GERD for 204 countries in 2021. ASIR, age-standardized incidence rate; ASPR, age-standardized prevalence rate; ASYR, age-standardized YLD rate; GBD, Global Burden of Disease; GERD, gastroesophageal reflux disease; SDI, socio-demographic index.

**Figure 3:**
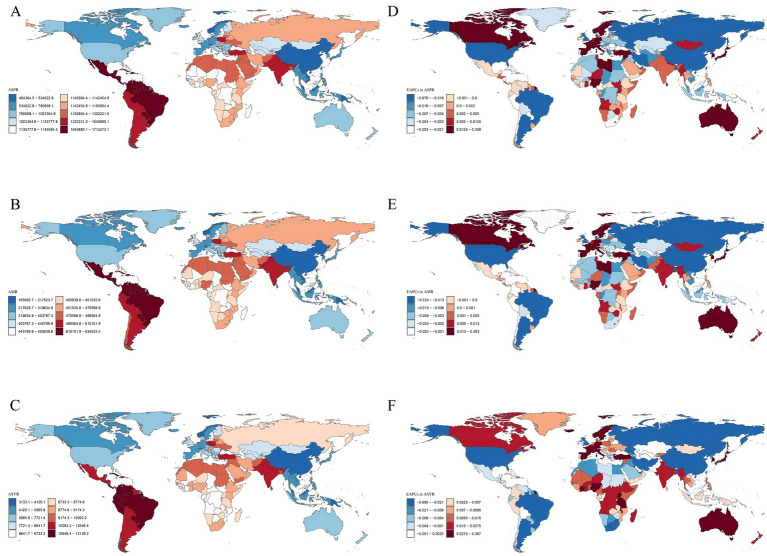
The global burden of GERD in 204 countries and territories. **(A)** ASPR of GERD (per 100,000 population) in 2021. **(B)** ASIR of GERD (per 100,000 population) in 2021. **(C)** ASYR due to GERD (per 100,000 population) in 2021. **(D)** EAPC in ASPR for GERD from 1990 to 2021. **(E)** EAPC in ASIR for GERD from 1990 to 2021. **(F)** EAPC in ASYR for GERD from 1990 to 2021. ASPR, age-standardized prevalence rate; ASIR, age-standardized incidence rate; ASYR, age-standardized years lived with disability; EAPC, estimated annual percentage change; GERD, gastroesophageal reflux disease. ASPR, age-standardized prevalence rate; ASIR, age-standardized incidence rate; ASYR, age-standardized years lived with disability; EAPC, estimated annual percentage change; GERD, gastroesophageal reflux disease.

There were significant differences in the GERD burden across 21 GBD regions. In 2021, the highest prevalence of cases, incident cases, and YLDs of GERD was observed in East Asia, North Africa, the Middle East, and South Asia among the GBD regions (Table 1; Figures 2A–C; Supplementary Tables 1–3). Conversely, the Caribbean, Australasia, and Oceania exhibited the lowest prevalence, incidence, and YLDs (Table 1; Figure 2; Supplementary Tables 1–3). Tropical Latin America recorded the highest ASPR of 16,681.34/100,000 (95% CI: 14,832.41 to 18,433.23), ASIR of 6,248.12/100,000 (95% CI: 5,567.61 to 6,874.8), and ASYR of 127.65/100,000 (95% CI: 64.69–225.33; Table 1; Supplementary Tables 1–3). East Asia exhibited the lowest ASPR of 4,554.18 (95% CI: 3,961.91–5,170.75), ASIR of 1,849.87/100,000 (95% CI: 1,609.66–2,090.47), and ASYR of 35.22/100,000 (95% CI: 17.68–62.55) among all regions. Over the last 30 years, ASPR and ASIR exhibited minimal variation in almost half of the regions, whereas the total prevalence, YLDs, and incidence consistently increased across all GBD regions during the study period (Supplementary Tables 1–3). From 1990 to 2021, the lowest EAPCs for ASPR, ASIR, and ASYR were recorded in high-income North America (−0.627, −0.483, and −0.644, respectively), whereas the highest was observed in the high-income Asia Pacific region (0.192, 0.159, and 0.199, respectively; Table 1; Figure 3).

### Trends in GERD burden at the national and regional levels

At both national and regional levels, there were significant differences in the GERD burden. In 2021, among the 204 countries, India and China recorded the highest number of prevalent cases [approximately 194 million (95% CI: 172.15 million–219.51 million) and 81.33 million (95% CI: 70.80 million–91.95 million), respectively], incident cases [approximately 76.32 million (95% CI: 67.27 million–84.68 million) and 32.39 million (95% CI: 27.85 million–36.71 million), respectively], and YLD cases [approximately 1.49 million (95% CI: 0.75 million–2.64 million) and 0.63 million (95% CI: 0.31 million–1.12 million), respectively] (Supplementary Tables 4–6). In 2021, Brazil recorded the highest ASIR at 6,249.93/100,000 (95% CI: 5,569.09–6,875.72), whereas Paraguay exhibited the highest ASPR and ASYR at 16,774.68/100,000 (95% CI: 14,835.88–18,709.59) and 128.91/100,000 (95% CI: 65.11–229.61), respectively (Figures 2D–F; Supplementary Tables 4–6). Conversely, Norway exhibited the lowest ASPR, ASIR, and ASYR at 4,330.94/100,000 (95% CI: 3,783.45–4,956.78), 1,838.34/100,000 (95% CI: 1,582.47–2,072.53), and 33.37/100,000 (95% CI: 16.99–60.04), respectively. From 1990 to 2021, ASPR significantly increased in South Korea (EAPC = 0.359), Turkey (EAPC = 0.274), and Taiwan (Province of China) (EAPC = 0.153); ASIR significantly increased in South Korea (EAPC = 0.263), Turkey (EAPC = 0.157), and Switzerland (EAPC = 0.152). For Mainland China, we found that the EAPC for ASPR and ASIR from 1990 to 2021 were −0.088 and −0.082, respectively, indicating a slight decrease in both ASPR and ASIR during this period. Furthermore, the most significant reductions in ASPR, ASIR, and ASYR were observed in the United States, Russia, and Argentina, with EAPCs of −0.676, −0.524, and −0.695, respectively (Supplementary Tables 4–6).

### Gender and age trends in GERD burden

**Figure 4:**
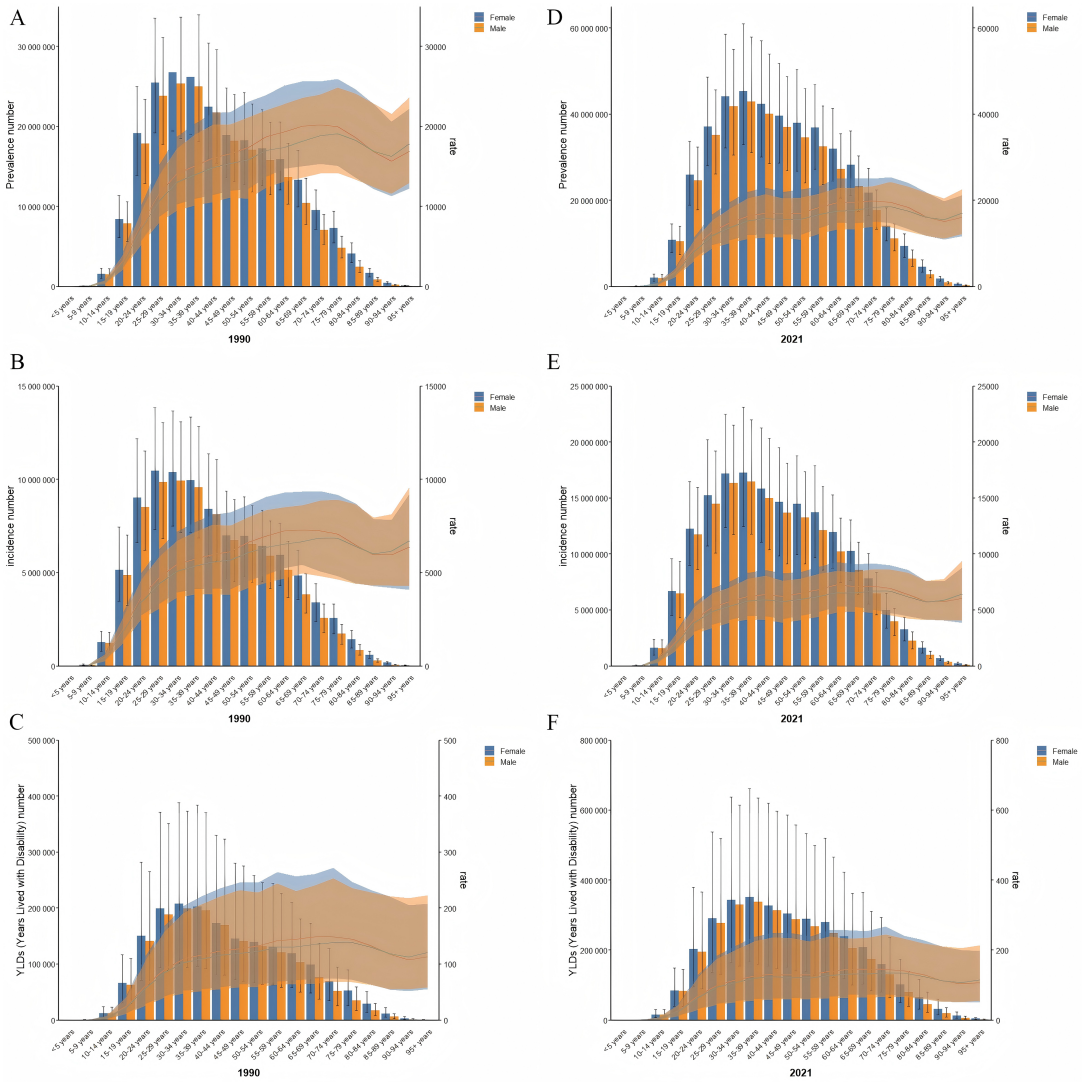
Age patterns by sex of the total number and age-specific prevalence rate **(A)**, incidence rate **(B)**, and YLD rate **(C)** for GERD in 1990, and age patterns by sex of the total number and age-specific prevalence rate **(D)**, incidence rate **(E)**, and YLD rate **(F)** for GERD in 2021. Error bars indicate the 95% uncertainty interval (UI) for the number of cases, and shading represents the 95% UI for the rates. GERD, gastroesophageal reflux disease. ASPR, age-standardized prevalence rate; ASIR, age-standardized incidence rate; ASYR, age-standardized years lived with disability; EAPC, estimated annual percentage change; GERD, gastroesophageal reflux disease.

A significant disparity in the GERD burden was observed between females and males, with females exhibiting a persistent trend of higher disease burden in comparison to males (Figure 4A–C). In 2021, females exhibited a higher prevalence, incidence, and YLDs than males worldwide. Female ASPR, ASIR, and ASYR also exceeded those of males (Figures 4D–F). Incident cases, prevalent cases, and YLDs were highest in the 35–39 age group (Figures 4D–F). ASPR for GERD was highest among males aged 75–79 and females aged 70–74 years, whereas ASYR peaked in the 70–74 age group for both genders. ASIR reached its highest levels in females aged 60–64 and males aged 70–74.

**Figure 5:**
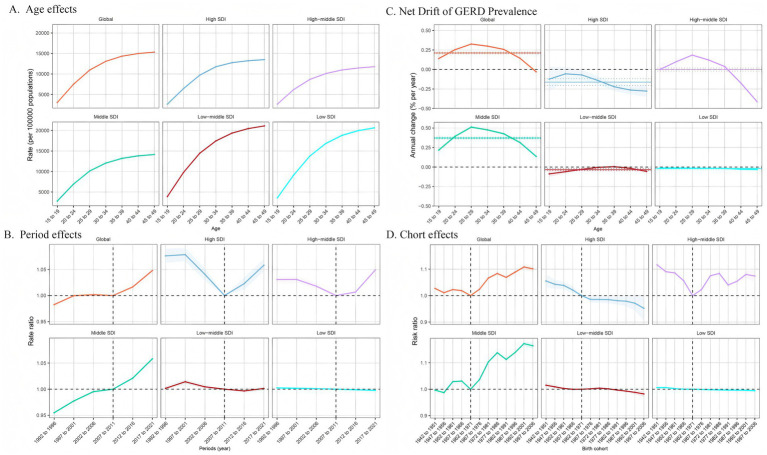
Age, period, and birth cohort effects on GERD prevalence among 15–49 year olds by APC models. **(A)** Age effect, longitudinal age-specific rates adjusted for cohort and period variations. **(B)** Period effect, relative risk of GERD prevalence between 1992 and 1996 and 2017–2021, with 1992–1996 as the baseline. **(C)** Local drift and age distribution of GERD prevalence from 1992 to 2021 across SDI quintiles. **(D)** Birth cohort effect, relative risk of prevalence, calculated as the ratio of rates from the 1942–1951 to the 1997–2006 cohort, with 1972–1981 as the reference. Dots and shaded areas represent rates or rate ratios with 95% CIs. ASPR, age-standardized prevalence rate; ASIR, age-standardized incidence rate; ASYR, age-standardized years lived with disability; EAPC, estimated annual percentage change; GERD, gastroesophageal reflux disease.

**Figure 6:**
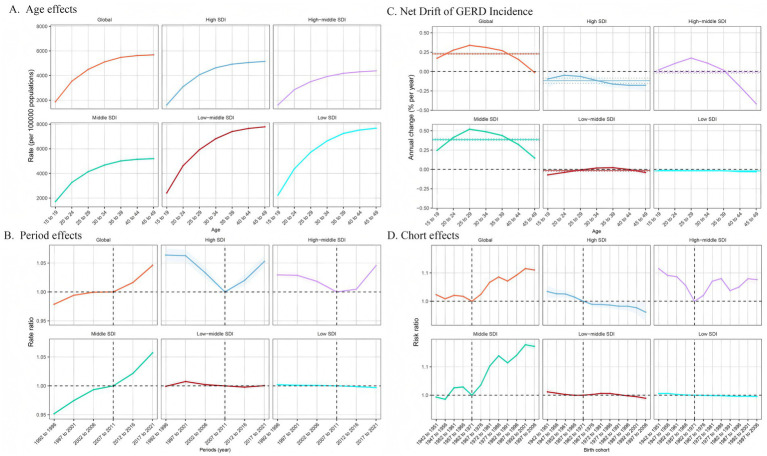
Age, period, and birth cohort effects on GERD incidence among 15–49 year olds by APC models. **(A)** Age effect: longitudinal age-specific rates adjusted for cohort and period variations. **(B)** Period effect: relative risk of GERD incidence between 1992–1996 and 2017–2021, with 1992–1996 as the baseline. **(C)** Local drift and age distribution of GERD incidence from 1992 to 2021 across SDI quintiles. **(D)** Birth cohort effect: relative risk of incidence, calculated as the ratio of rates from the 1942–1951 to the 1997–2006 cohort, with 1972–1981 as the reference. Dots and shaded areas represent rates or rate ratios with 95% CIs. ASPR, age-standardized prevalence rate; ASIR, age-standardized incidence rate; ASYR, age-standardized years lived with disability; EAPC, estimated annual percentage change; GERD, gastroesophageal reflux disease.

**Figure 7:**
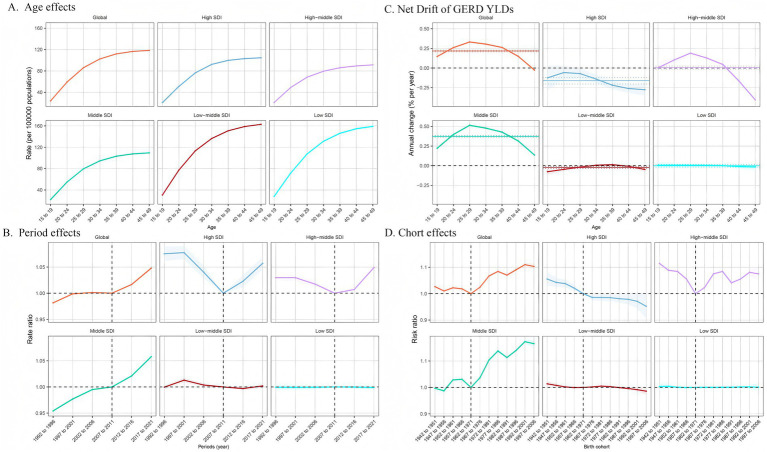
Age, period, and birth cohort effects on GERD YLDs among 15–49 year olds by APC models. **(A)** Age effect: longitudinal age-specific rates adjusted for cohort and period variations. **(B)** Period effect: relative risk of GERD YLDs between 1992–1996 and 2017–2021, with 1992–1996 as the baseline. **(C)** Local drift and age distribution of GERD YLDs from 1992 to 2021 across SDI quintiles. **(D)** Birth cohort effect: relative risk of YLDs, calculated as the ratio of rates from the 1942–1951 to the 1997–2006 cohort, with 1972–1981 as the reference. Dots and shaded areas represent rates or rate ratios with 95% CIs. ASPR, age-standardized prevalence rate; ASIR, age-standardized incidence rate; ASYR, age-standardized years lived with disability; EAPC, estimated annual percentage change; GERD, gastroesophageal reflux disease.

Figure 5C illustrates the annual percentage change in GERD prevalence for individuals aged 15–49. Globally, GERD prevalence displayed a distinctive trend, characterized by a decline in the 45–49 age group, in contrast to the upward trend observed in other age groups, particularly the significant rises in the 25–29 and 30–34 age groups. In the low- and low-middle SDI regions, the overall prevalence trend was downward with relatively stable changes. In the middle SDI regions, all age demographics exhibited an upward trend, peaking in the 25–29 age group. In high-middle SDI regions, prevalence increased until age 35–39 and then began to decline. In high SDI regions, the net drift of GERD prevalence remained relatively stable and displayed a downward trend. Figures 6C, 7C illustrate the net drift in GERD incidence and YLDs for the 15–49 age group, respectively.

### Impact of age, period, and birth cohort on GERD burden in the 15–49 age group

Figure 5 demonstrates the effects of age, period, and cohort on GERD prevalence in the 15–49 age group based on the APC model. This age group was selected because of the global trend of GERD affecting the younger population. Overall, age impacts displayed comparable trends across SDI regions, with the 45–49 age group demonstrating the highest risk, which decreased in younger age groups. The prevalence rates in the high-middle SDI region were lower across all age groups, with minimal variation between age groups (Figure 5A). Figures 6A, 7A show age effects on GERD incidence and YLDs, respectively. Figure 5B depicts the period effects on GERD prevalence from 1992 to 2021 in the 15–49 age group, expressed as relative risk (Rate Ratio). Recently, the prevalence has risen in both global and middle SDI regions, particularly between 2012 and 2021. In the high and high-middle SDI regions, the risk was lowest from 2007 to 2011 but subsequently increased, while prevalence remained relatively stable in the low-middle and low SDI regions (Figures 6B, 7B for incidence and YLD period effects, respectively). Figure 5D displays the effects of birth cohort on GERD prevalence in the 15–49 age group globally and across SDI regions, revealing a downward trend reaching a low point in the 1962–1971 cohort, followed by a gradual increase. The trend in the middle SDI region reflects a global pattern, whereas the high SDI regions exhibit a declining trend, particularly in cohorts born after 1967. In high-middle SDI regions, the lowest risk was observed in the 1962–1971 cohort, with subsequent cohorts demonstrating a comparatively higher risk. The low-middle and low SDI regions displayed stable risk across birth cohorts, indicating gradual improvement (Figures 6D, 7D for incidence and YLD cohort effects).

### Global disease burden prediction for GERD among 15–49 age group to 2035

**Figure 8:**
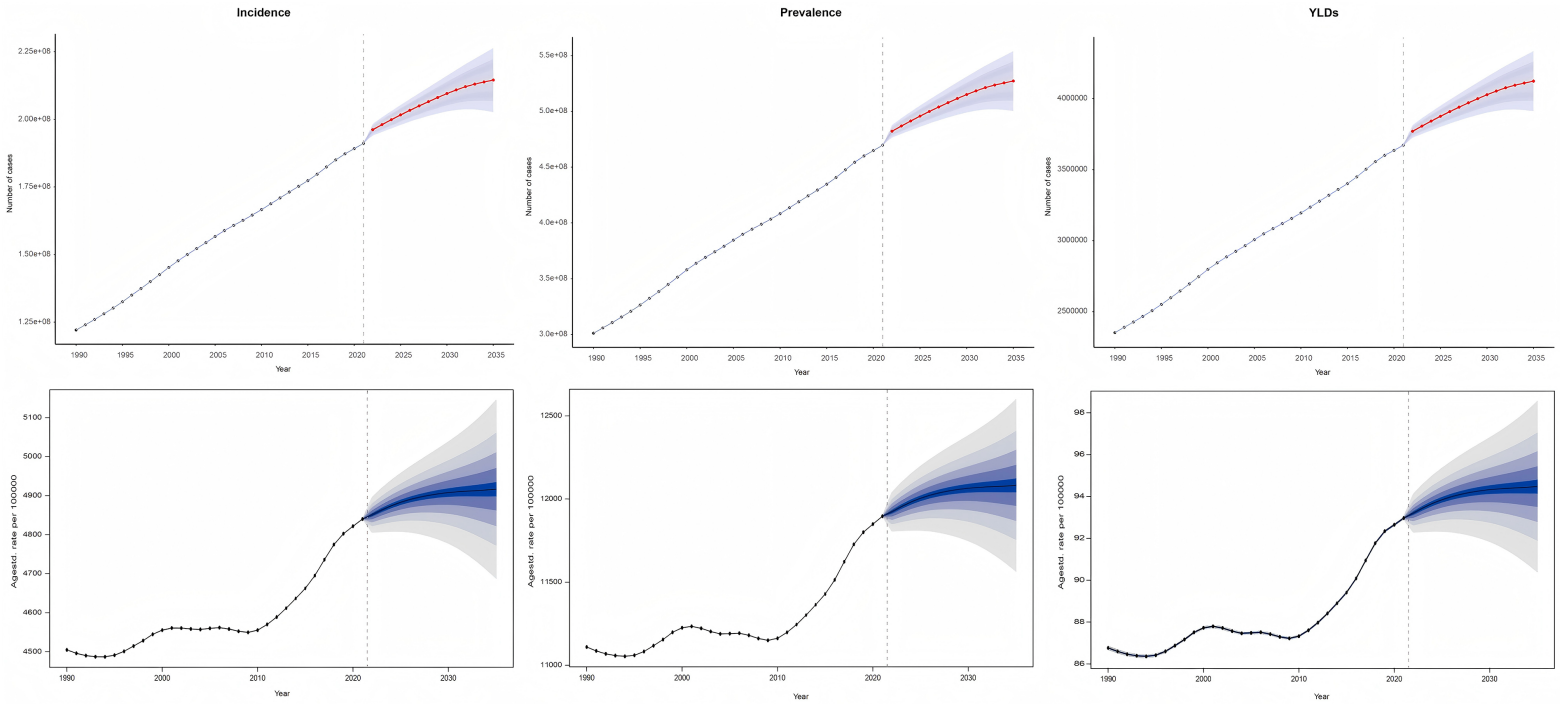
Future forecast of global ASIR, ASPR and ASYR of GERD. ASPR, age-standardized prevalence rate; ASIR, age-standardized incidence rate; ASYR, age-standardized years lived with disability; EAPC, estimated annual percentage change; GERD, gastroesophageal reflux disease.

The BAPC model was used to project future trends in GERD burden from 2022 to 2035, specifically focusing on the 15–49 age group (Figure 8). By 2035, the total number of GERD cases globally within this age group is projected to reach approximately 527.24 million (95% UI: 500.38 million–554.09 million), with an ASPR of 12,082.06/100,000. The total incidence is estimated to be 214.56 million (95% UI: 202.67 million–226.44 million), with an ASIR of 4,916.68/100,000. YLDs are expected to reach approximately 4.12 million (95% UI: 3.91 million–4.33 million), while the ASYR is forecasted to be 94.47/100,000 by 2035. These projections underscore the ongoing health burden posed by GERD on a global scale, with rising prevalence and incidence highlighting the need for targeted management and prevention strategies within the 15–49 age demographic, if current trends continue.

## Discussion

This study utilized GBD 2021’s publicly available modeling data and methodology to systematically analyze the last 32-year trends in GERD burden across 204 countries and territories. It provides the latest comprehensive data on global GERD prevalence, incidence, and YLDs from 1990 to 2021. To deepen our understanding, we applied the APC model to specifically examine the influence of age, period, and birth cohort on GERD trends in younger populations across different SDI regions. Besides, we employed the BAPC predictive model to forecast the GERD burden in 2035. This study provides valuable insights for global healthcare policy planning and resource allocation aimed at mitigating the growing global GERD burden.

Previous studies have reported on the burden of GERD (3, 5, 8). Globally, the GBD 2017 demonstrated that the ASPR and ASYR remained stable from 1990 to 2017, but it did not estimate the ASIR trend. The GBD 2019 indicated that ASPR remained stable during this period, whereas ASYR and ASIR showed an increase. The GBD 2021 significantly improved methodology and data coverage, employing more advanced statistical models to enhance the accuracy of estimates in regions with low data quality. Our study found that the global burden of GERD rose markedly over the past 32 years. This upward trend may be attributed to lifestyle changes, including sedentary behavior, poor diet, and rising smoking and alcohol consumption (9, 10, 17). Moreover, increased obesity rates elevate abdominal pressure, promoting reflux (18). Population aging further exacerbates GERD risk due to age-related decline in lower esophageal sphincter function (19). Future studies should investigate interactions between these factors and evaluate potential public health interventions to mitigate their impact on GERD prevalence.

Significant differences in the GERD burden were found across regions with varying SDI levels. High SDI regions typically exhibit lower ASPR, ASIR and ASYR, which are closely related to their comprehensive primary healthcare systems and robust chronic disease management networks. These systems facilitate early screening (including endoscopy) and long-term follow-up, which effectively mitigate GERD severity (8). Additionally, high-SDI regions include medications like proton pump inhibitors in their healthcare coverage, improving treatment adherence and reducing the occurrence and progression of GERD (20). Conversely, fragmented healthcare resources in low-SDI regions often limit care to acute episodes. This lack of continuous management contributes to disease recurrence and progressive worsening (21, 22). Economic constraints mean that residents in these regions often consume inexpensive, high-fat, high-sugar diets and pursue physically demanding occupations. These are both factors that elevate intra-abdominal pressure and increase the risk of reflux (18). Compounding these challenges, irregular work schedules and overcrowded living conditions further exacerbate GERD pathogenesis (23). Low-SDI regions frequently enter a ‘disease burden-resource allocation’ vicious cycle: limited funding prioritizes infectious disease control while chronic conditions like GERD remain neglected (22). By contrast, high SDI countries employ multi-level GERD prevention strategies, including nutritional programs and public health initiatives, that effectively reduce disease incidence (24). Therefore, public health policies should focus on low SDI regions, improving healthcare infrastructure, strengthening health education, and promoting scientifically-based lifestyle interventions to alleviate the GERD burden in these areas.

Globally, there are significant differences in the GERD burden across different GBD regions. It is estimated that the prevalence, incidence, and YLDs of GERD are highest in East Asia, North Africa, the Middle East, and South Asia. In East Asia, rapid urbanization has driven a dietary transition, blending traditional eating patterns with Western-style diets. Notably, the combined effects of high-sodium traditional foods (e.g., cured meats, pickled vegetables, and salt-rich soups) and increased consumption of saturated fats from processed fast foods have contributed to escalating obesity rates, a key driver of GERD (25). The Middle East exhibits distinct dietary risk factors, particularly the prevalent use of reflux-inducing spices like chili and cumin in traditional cuisine, which may exacerbate GERD symptoms (26). South Asia and North Africa primarily face challenges related to the unequal distribution of healthcare resources, including uneven endoscopic screening coverage in resource-limited areas, inadequate training of primary care physicians, and low health literacy, which leads to many GERD cases being misdiagnosed as common indigestion, thereby delaying treatment (27, 28). This global health inequality needs to be addressed through the fair distribution of medical resources, the formulation of targeted prevention strategies, and strengthening medical technology exchanges and cooperation between regions.

Our study further revealed that women globally exhibit higher GERD prevalence, incidence, and YLDs than men, consistent with previous findings (7, 12). Eusebi et al. observed regional differences in GERD’s association with gender and significantly higher burdens among women in the Middle East, North Africa, East Asia, and South Asia (7). This gender disparity may be related to physiological factors, hormonal fluctuations, and physiological changes during pregnancy. Women have weaker lower esophageal sphincter function, and hormonal changes may increase the risk of gastroesophageal reflux (29). Additionally, women are more likely to report GERD symptoms and seek treatment, leading to higher diagnosis rates. Therefore, future research should explore the impact of gender differences on the pathogenesis of GERD, particularly in high-burden regions, and develop personalized prevention and treatment strategies for women.

This study also revealed differences in the GERD burden across different age groups. The GERD prevalence, incidence, and YLDs are highest in the 35–39 age group, suggesting that this age range may represent a peak period for GERD. A study conducted by Yamasaki similarly revealed a notable increase in the proportion of GERD patients in the 30–39 age group (12). Additionally, the older adult population also show higher ASPR and ASYR. Middle-aged and young adults often face significant work-related stress, and unhealthy lifestyle habits such as irregular eating patterns, high-fat diets, and alcohol consumption are major triggers for GERD (6). As individuals age, the decline in lower esophageal sphincter function and the presence of other chronic conditions (such as diabetes) further increase GERD risk in the older adult population (12). Therefore, intervention strategies for different age groups should be differentiated, particularly in terms of lifestyle and chronic disease management.

This research is the first to implement an APC model at global and SDI levels, focusing on GERD prevalence, incidence, and YLD trends among the 15–49 age group. It offers a comprehensive comparison across SDI regions, refining insights into the GERD burden among younger populations. Delshadet et al. discovered that the incidence of GERD symptoms increased with age until a specific point, after which the risk declined. Compared with individuals aged 18–29, those aged 30–49 are more likely to exhibit severe GERD symptoms as described by the Montreal criteria. However, for those aged 60 and above, the prevalence of such symptoms was comparatively lower (2). By analyzing age, period, and cohort effects, this research identifies increasing burdens in younger groups, especially the 25–34 age group. Unfavorable period effects were particularly significant in high-middle and high-SDI regions, reflecting rising prevalence over time, while adverse cohort effects were particularly evident in middle and high-middle SDI regions, suggesting an increasing burden among more recent birth cohorts. These trends underscore the intricate interplay of age, temporal, and generational factors in shaping GERD’s global burden. Projections for 2035 suggest a continued rise in the GERD burden, with global cases expected to reach approximately 527 million, underlining the need for targeted interventions, particularly in regions with limited healthcare resources and younger age groups facing lifestyle changes.

This study has some limitations. First, the GBD database relies on healthcare system reports from various countries, which may lead to a systematic underestimation of GERD, especially in low- and middle-income regions (11). Some GERD patients are not recorded due to self-medication or failure to seek medical care, and diagnostic standard differences (e.g., insufficient endoscopy coverage) further affect the comparability of the data (6). The GBD addresses data scarcity through a hierarchical Bayesian model, flexibly borrowing data from surrounding countries, but these estimates may not fully represent the actual situation in the target region. Second, although GBD is the most authoritative global disease burden database, its integrated modeling methods, while scientifically rigorous, still introduce uncertainties in the prediction of GERD. Additionally, the capture rates for acute diseases (e.g., myocardial infarction) and infectious diseases (e.g., COVID-19) are generally higher than those for chronic conditions, including GERD, while other chronic diseases and mental health disorders are also underreported (11). Although GBD improves estimation accuracy through data calibration, the YLDs value for GERD does not fully reflect its long-term impact on quality of life. Future research could consider using quality-adjusted life years combined with symptom frequency and economic burden for a more comprehensive evaluation (11). Furthermore, incorporating more raw data and long-term cohort studies would help more accurately assess GERD’s trends and disease burden. Our research standards align with expert consensus, and the data is consistent with prior research, demonstrating the validity of our study.

Overall, with the continuous increase in global GERD prevalence, incidence, and YLDs, GERD remains a significant contributor to the economic burden. Due to its detrimental effects on patients’ quality of life, its correlation with an elevated risk of esophageal adenocarcinoma, and the potential side effects of proton pump inhibitors used in treatment, additional investigation is needed (30–32). Future studies should focus on long-term cohort research to explore the factors, such as lifestyle, dietary habits, and psychological stress, contributing to the increased GERD burden in the 25–34 age group, and further investigate the pathophysiological mechanisms in younger populations. Public health strategies should focus on improving healthcare access in low-income and resource-poor regions, promoting early screening and treatment of GERD, and developing personalized interventions tailored to different age groups and genders to reduce the GERD burden.

## Conclusion

Based on GBD 2021 data, this study conducted a rigorous and systematic analysis of the 32-year trends in GERD burden across global, regional, and national levels, revealing a sustained increase, particularly focusing on younger populations. These results underscore the urgent need to develop targeted, evidence-based prevention and intervention strategies in high-risk regions and younger age groups to effectively address the escalating global public health challenges associated with GERD.

## Data Availability

The original contributions presented in the study are included in the article/Supplementary material, further inquiries can be directed to the corresponding authors.

## Footnotes

^1^ https://vizhub.healthdata.org/gbd-results/

## Supplementary material

SUPPLEMENTARY TABLE 1 Prevalence of GERD in 1990 and 2021 across global, five SDI regions, and 21 GBD regions, with the EAPC from 1990 to 2021.

SUPPLEMENTARY TABLE 2 Incidence of GERD in 1990 and 2021 across global, five SDI regions, and 21 GBD regions, with the EAPC from 1990 to 2021.

SUPPLEMENTARY TABLE 3 YLDs due to GERD in 1990 and 2021 across global, five SDI regions, and 21 GBD regions, with the EAPC from 1990 to 2021.

SUPPLEMENTARY TABLE 4 Prevalence of GERD in 1990 and 2021 across 204 countries and territories, with the EAPC from 1990 to 2021.

SUPPLEMENTARY TABLE 5 Incidence of GERD in 1990 and 2021 across 204 countries and territories, with the EAPC from 1990 to 2021.

SUPPLEMENTARY TABLE 6 YLDs due to GERD in 1990 and 2021 across 204 countries and territories, with the EAPC from 1990 to 2021.
