# Supplementary material for: Global, regional, and national burden of gastroesophageal reflux disease (1990–2021): age-period-cohort analysis and Bayesian projections
Source: Front Public Health. 2025 Jul 9;13:1576527. doi: 10.3389/fpubh.2025.1576527 (PMC12283765; doi:10.3389/fpubh.2025.1576527)
Supplement: Supplementary file 7 [file Data_Sheet_1.pdf]

Checklist of information that should be included in new reports of global health estimates

Item #	Checklist item	Reported on page #
**Objectives and funding**		
**1**	Define the indicator(s), populations (including age, sex, and geographic entities), and time period(s) for which estimates were made.	Manuscript (Methods)
**2**	List the funding sources for the work.	Manuscript (Funding)
**Data Inputs**		
For all data inputs from multiple sources that are synthesized as part of the study:		
**3**	Describe how the data were identified and how the data were accessed.	Manuscript (Methods)
**4**	Specify the inclusion and exclusion criteria. Identify all ad-hoc exclusions.	Manuscript (Methods)
**5**	Provide information on all included data sources and their main characteristics. For each data source used, report reference information or contact name/institution, population represented, data collection method, year(s) of data collection, sex and age range, diagnostic criteria or measurement method, and sample size, as relevant.	https://ghdx. healthdata.org/gbd-2021/sources
**6**	Identify and describe any categories of input data that have potentially important biases (e.g., based on characteristics listed in item 5).	Manuscript (Methods)
For data inputs that contribute to the analysis but were not synthesized as part of the study:		
**7**	Describe and give sources for any other data inputs.	https://ghdx.healthdata. org/gbd-2021/sources
For all data inputs:		
**8**	Provide all data inputs in a file format from which data can be efficiently extracted (e.g., a spreadsheet rather than a PDF), including all relevant meta-data listed in item 5. For any data inputs that cannot be shared because of ethical or legal reasons, such as third-party ownership, provide a contact name or the name of the institution that retains the right to the data.	Online data visualization tools, data query tools, and the Global Health Data Exchange http://ghdx.healthdata .org/gbd-data-tool
**Data analysis**		
**9**	Provide a conceptual overview of the data analysis method. A diagram may be helpful.	Manuscript (Methods)
**10**	Provide a detailed description of all steps of the analysis, including mathematical formulae. This description should cover, as relevant, data cleaning, data pre-processing, data adjustments and weighting of data sources, and mathematical or statistical model(s).	Manuscript (Methods)
**11**	Describe how candidate models were evaluated and how the final model(s) were selected.	Manuscript (Methods)
**12**	Provide the results of an evaluation of model performance, if done, as well as the results of any relevant sensitivity analysis.	Manuscript (Methods)
**13**	Describe methods for calculating uncertainty of the estimates. State which sources of uncertainty were, and were not, accounted for in the uncertainty analysis.	Manuscript (Methods)
**14**	State how analytic or statistical source code used to generate estimates can be accessed.	Code is provided at https://ghdx.healthdata. org/c
**Results and Discussion**		
**15**	Provide published estimates in a file format from which data can be efficiently extracted.	Manuscript, supplementary results, and online data tools
**16**	Report a quantitative measure of the uncertainty of the estimates (e.g. uncertainty intervals).	Manuscript(Results and Disc ussion), supplementary resul ts, and online data tools
**17**	Interpret results in light of existing evidence. If updating a previous set of estimates, describe the reasons for changes in estimates.	Manuscript(Results and Disc ussion), supplementary resul ts, and online data tools
**18**	Discuss limitations of the estimates. Include a discussion of any modelling assumptions or data limitations that affect interpretation of the estimates.	Manuscript (Limitation)

This checklist should be used in conjunction with the GATHER statement and Explanation and Elaboration document, found on gather-statement.org
